# Atypical Presentation of Behçet Disease Unmasked by Acute Coronary Syndrome

**DOI:** 10.1016/j.jaccas.2025.104663

**Published:** 2025-08-20

**Authors:** Mahmoud Gomaa, Osama Elshaer

**Affiliations:** a Wexner Medical Center, The Ohio State University, Columbus, Ohio, USA; b Department of Cardiovascular Medicine, Kafrelsheikh University Hospital, Kafrelsheikh, Egypt

**Keywords:** acute coronary syndrome, atypical presentation, Behçet disease, coronary aneurysms, HLA-B51, vasculitis

## Abstract

**Background:**

Behçet disease is a rare multisystem vasculitis that may affect the coronary arteries, presenting as acute coronary syndrome (ACS) in young patients without risk factors.

**Case Summary:**

A 26-year-old man with recurrent oral ulcers experienced chest pain over 3 months. Coronary angiography revealed multivessel disease, and a subsequent angiogram showed coronary aneurysms, suggesting vasculitis. Behçet disease was diagnosed based on the International Criteria for Behçet's Disease and HLA-B51 positivity. The patient improved with corticosteroids and colchicine.

**Discussion:**

In young patients presenting with ACS and no significant risk factors for atherosclerosis, Behçet disease should be considered as a potential cause of coronary artery vasculitis. Coronary aneurysms may be an associated finding.

**Take-Home Messages:**

Vasculitis should be considered in the differential diagnosis for young patients with ACS without significant cardiovascular risk factors. Behçet disease can lead to coronary artery aneurysms; early recognition and management are critical.

## Visual Summary

**Figure:**
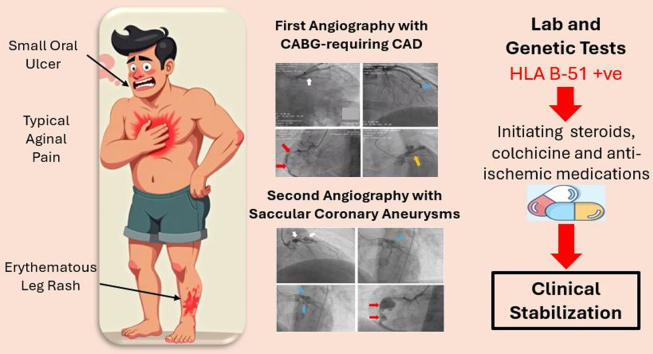

**Visual Summary:**
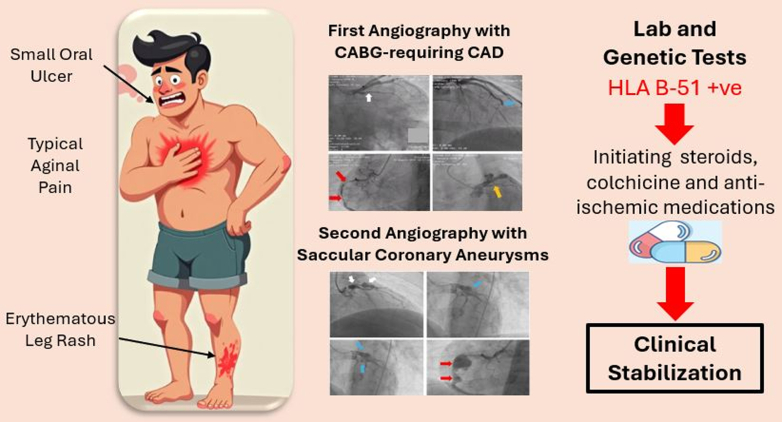
From Chest Pain to Behçet Diagnosis

## Personal and Past Medical History

The patient was a 26-year-old White male accountant; a nonsmoker with no history of alcohol or illicit drug intake. Past medical history was significant for recurrent oral ulcers since adolescence, tender erythematous skin lesions (erythema nodosum) on both legs, and a single episode of sudden unexplained visual loss in the right eye 2 years earlier, which was diagnosed as a transient ischemic attack after brain computed tomography was found to be normal. He had no first- or second-degree family members who had been diagnosed with genetic, rheumatologic, or immunologic diseases. There was also no family history of hyperlipidemia, premature atherosclerosis, or myocardial infarction.

**Take-Home Messages**

• Vasculitis is an important differential diagnosis for young patients presenting with acute coronary syndrome in the absence of significant cardiovascular risk factors for atherosclerosis.
• Behçet disease is a known cause of multisystem vasculitis that may lead to rare complications such as coronary artery aneurysms. Early recognition and management are essential, particularly in patients of Mediterranean descent.

## History of Presentation

**Figure 1:**
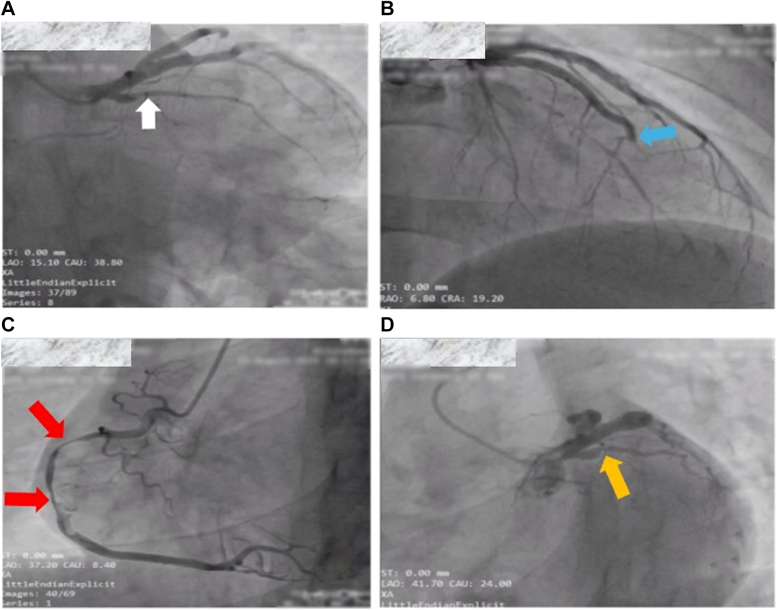
Different Views of the First Coronary Angiography With Multivessel Lesions (A) Right anterior oblique caudal view showing proximal subtotal left circumflex artery occlusion (white arrow). (B): Right anterior oblique cranial view showing total midsegment left anterior descending artery occlusion (blue arrow). (C) Left anterior oblique cranial view showing proximal to midsegment right coronary artery long subtotal occlusions (red arrows). (D) Left anterior oblique caudal view showing the proximal subtotal left circumflex artery occlusion (orange arrow).

**Figure 2:**
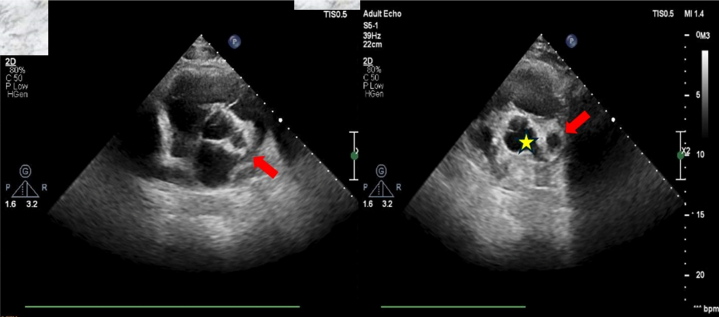
Parasternal Short-Axis View on Transthoracic Echocardiography Before Second Coronary Angiography Echolucent space (red arrows) next to the right coronary cusp of the aortic valve (star) suggests aneurysmal dilatation.

**Figure 3:**
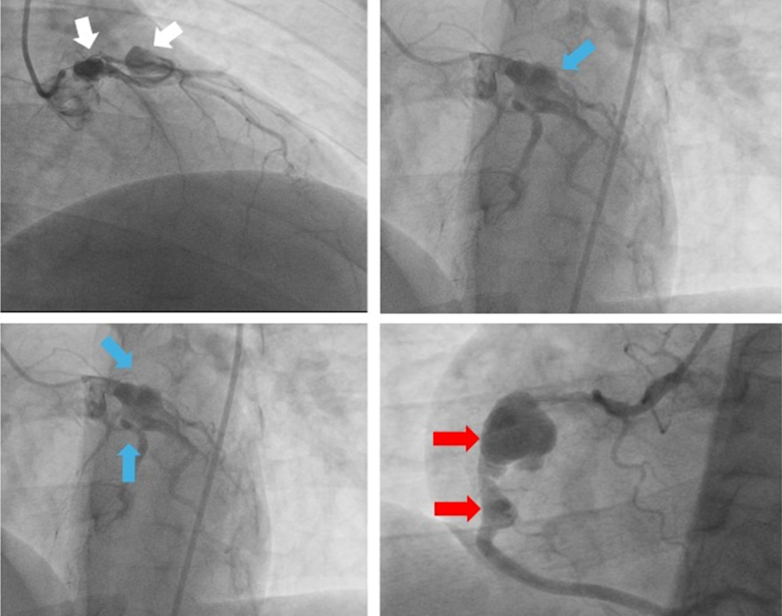
Saccular Coronary Artery Aneurysms on Different Views During Second Coronary Angiography Multiple saccular aneurysms in left system arteries on the right anterior oblique cranial view (white arrows) and right anterior oblique caudal view (blue arrows), and large saccular aneurysms in the proximal and midsegment right coronary artery (red arrows).

Symptoms started 3 months before presentation at our center with occasional retrosternal burning pain, for which he was prescribed an antacid after having a normal electrocardiogram. Two months later, he developed sudden severe crushing chest pain referring to both shoulders after a stressful emotional encounter. Electrocardiogram then revealed T-wave inversion in the inferior and lateral chest leads, and transthoracic echocardiography (TTE) showed preserved systolic function with no wall motion abnormalities. Subsequent coronary angiography on same day was significant for multivessel coronary artery disease, necessitating a coronary artery bypass (CAB) procedure (Figure 1). He was referred to us 1 week later to prepare for CAB and was put on tirofiban infusion, atorvastatin 80 mg, nitroglycerin, and intravenous morphine. Notably, an echolucent space next to the aortic valve was observed on his new TTE (Figure 2). After counseling with his family, he refused to consent for CAB and decided to proceed with coronary artery stenting (primary coronary intervention) of the doable arteries after discussion on the potential short- and long-term risks of his preference. Proceeding with his second angiography, several saccular aneurysms were observed, mainly affecting the proximal segments of the left anterior descending (LAD) artery, the left circumflex artery, and the right coronary artery and hindering the initiation of primary coronary intervention (Figure 3).

## Differential Diagnosis

The 2 main differential diagnoses were: 1) iatrogenic cause leading to coronary artery *Staphylococcus aureus* infection from his prior angiography; and 2) vasculitis. Infection was excluded after having negative pan-cultures, especially as there were no clinical or laboratory signs of septicemia except for a mildly elevated C-reactive protein level of 9 mg/L.

## Investigations

**Figure 4:**
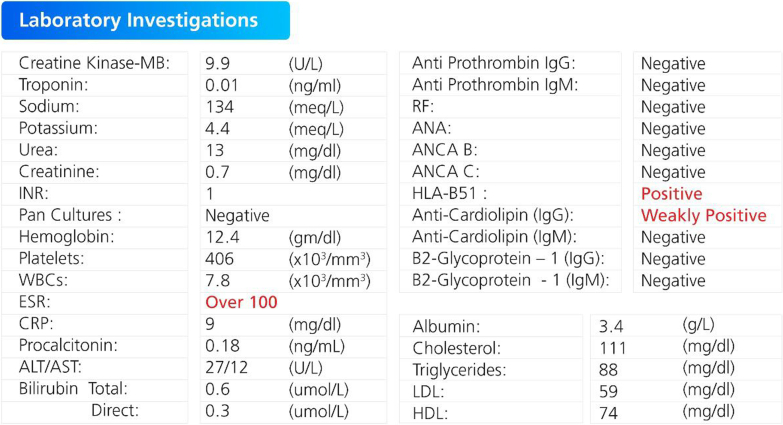
Significant Laboratory Investigations ALT/AST = alanine transaminase/aspartate transaminase; ANA = antinuclear antibody; ANCA = antineutrophil cytoplasmic antibodies; CRP = C-reactive protein; ESR = erythrocyte sedimentation rate; HDL = high-density lipoprotein cholesterol; INR = international normalized ratio; LDL = low-density lipoprotein cholesterol; MB = myocardial band; RF = rheumatoid factor; WBC = white blood cell.

The workup for vasculitis included serologic quantification of rheumatologic antibodies (rheumatoid factor, anti–double-stranded DNA antibodies, antinuclear antibody, antineutrophil cytoplasmic antibodies B and C, and antiphospholipid and anticardiolipin antibodies) and genetic testing (HLA subtyping). Results showed a weakly positive anticardiolipin titer and a positive HLA-B51 gene (Figure 4).

## Management

After consulting with rheumatology, a diagnosis of Behçet disease was considered, and the patient was placed on methylprednisolone 1 gram/d for 3 days, then switched to oral prednisolone 60 mg/d, azathioprine 50 mg 3 times per day, and colchicine 0.5 mg/d. Full anti-ischemic medications were continued as well. After the first week, the patient noticed gradual reduction in chest pain (from 9/10 to 2/10 on the Mankoski pain scale).

## Outcome

At 2 weeks, the patient was discharged with maintenance prednisone, azathioprine, and colchicine, and at the 1-month follow-up, he reported compliance with therapy, experiencing none of his previous symptoms during this period. Subsequent follow-up over the next several weeks included electrocardiography, which showed no new ischemic changes, and TTE, which showed preserved myocardial function and wall motion. However, coronary artery dilatation consistent with earlier imaging was still the same, without evidence of progression or new aneurysmal lesions. The patient is reconsidering CAB, and further evaluation—including the possibility of repeat coronary angiography—is currently under discussion.

## Discussion

Atherosclerotic CAD is the most prevalent cause of ACS. However, nonatherosclerotic causes such as vasculitis can also lead to CAD and should be considered in the differential diagnosis, especially in young patients. Behçet disease, a rare multisystem inflammatory disorder, causes vasculitis that can affect various body systems, and it often poses a diagnostic challenge because of its diverse manifestations. Its cardiac involvement is mostly pericardial, myocardial, or valvular.^1^ Coronary artery involvement in Behçet disease is rarely reported, yet it is associated with significant morbidity and mortality.^2^

Here, we presented a case of ACS that was challenging both in diagnosis and management. Diagnosis was made after the patient achieved a score of 4 based on the International Criteria for Behçet's Disease: history of recurrent oral ulcers (2 points), erythema nodosum (1 point), and central nervous system involvement (1 point). Whereas the past incident of temporary vision loss was previously assumed to be an idiopathic transient ischemic attack, it could be attributed to associated vasculitis of Behçet disease, particularly considering the absence of the common risk factors for cerebrovascular incidents.^3^ This is supported by HLA B51 positivity. The saccular appearance of the coronary arteries was the main finding that led us to consider Behçet disease, which had not been previously diagnosed. Fortunately, there were no residual effects on the patient’s cardiac function or complications from the ACS after the timely initiation of corticosteroids and colchicine.

Because Behçet disease can cause inflammation of the blood vessels, increasing the risk of aneurysm formation and thrombosis, cardiovascular involvement is a logical expectation. However, studies have indicated that only 7% to 46% of patients with Behçet disease experience cardiovascular manifestations, with mortality rates up to 20% in those with severe vascular involvement.^4^ Additionally, it is believed that younger patients with cardiac complications may be more prevalent in the Mediterranean basin.^5^ A recent study identified male sex, smoking, and papulopustular lesions as factors associated with cardiovascular involvement in Behçet disease.^6^

Because of the rarity of the condition, cardiovascular consequences of Behçet disease in the literature have frequently been documented as case reports with diverse presentations. For example, Yahalom et al reported 2 cases: a 65-year-old man with Behçet disease who required a permanent pacemaker owing to complete heart block; the implantation was unsuccessful because of old obstructions in the upper thoracic veins. The remaining case involved a 22-year-old man with severe dyspnea and a chest x-ray initially suggesting bilateral pneumonia, which was later diagnosed as pulmonary embolism with a large thrombus in the right atrium, secondary to previously undiagnosed Behçet disease.^7^

In reviewing cases of ACS, we found a similar case reported by a Turkish center: a 29-year-old man with known Behçet disease who presented with ACS and who underwent coronary angiography, which revealed a fresh thrombus in the proximal right coronary artery, an aneurysm in the proximal LAD artery, and a significant lesion after the aneurysm. Intravenous tirofiban was administered, leading to thrombus dissolution in the right coronary artery, but the LAD artery aneurysm grew, and a new aneurysm appeared in the right coronary artery.^8^

When ACS is encountered in a young patient with no family history or significant risk factors, coronary artery anomalies and inherited or acquired hypercoagulable conditions are the first considerations in the differential.^9^ Moreover, even when immunologic causes are considered, medium-vessel vasculitides such as polyarteritis nodosa and Kawasaki disease are reported in the literature to have the highest coronary involvement (up to 50% and 20%, respectively).^10^ Behçet disease is often overlooked despite affecting various types of blood vessels, and ongoing research aims to investigate the vasculitis pathogenesis and how to predict the potentially affected vessels—including the coronaries—based on individual genetic and acquired factors.^11^

## Conclusions

The management of ACS in young patients without significant cardiovascular risk factors should consider Behçet disease as a potential cause of coronary artery vasculitis, particularly in its most prevalent region, the Mediterranean basin. Associated saccular coronary aneurysms may also be a potential finding.

### Data Availability

The data in this case report, including the angiographic images, are available from the corresponding author on reasonable request.

## Funding Support and Author Disclosures

The authors have reported that they have no relationships relevant to the contents of this paper to disclose.
